# The Hydrophilic Metabolite UMP Alleviates Obesity Traits through a HIF2α‐ACER2‐Ceramide Signaling Axis

**DOI:** 10.1002/advs.202309525

**Published:** 2024-03-09

**Authors:** Huiying Liu, Pengcheng Wang, Feng Xu, Qixing Nie, Sen Yan, Zhipeng Zhang, Yi Zhang, Changtao Jiang, Xiaomei Qin, Yanli Pang

**Affiliations:** ^1^ Department of Physiology and Pathophysiology School of Basic Medical Sciences State Key Laboratory of Vascular Homeostasis and Remodeling Peking University Beijing 100191 China; ^2^ Center for Obesity and Metabolic Disease Research School of Basic Medical Sciences Peking University Beijing 100191 China; ^3^ Center of Basic Medical Research Institute of Medical Innovation and Research Third Hospital Peking University Beijing 100191 China; ^4^ Clinical Pharmacology and Pharmacometrics Janssen China Research & Development Beijing 100191 China; ^5^ State Key Laboratory of Food Science and Resources China‐Canada Joint Lab of Food Science and Technology Key Laboratory of Bioactive Polysaccharides of Jiangxi Province Nanchang University Nanchang 330013 China; ^6^ Center for Reproductive Medicine Department of Obstetrics and Gynecology State Key Laboratory of Female Fertility Preservation and Promotion Peking University Third Hospital Beijing 100191 China; ^7^ General Surgery Department Third Hospital Peking University Beijing 100191 China; ^8^ Department of Immunology School of Basic Medical Sciences NHC Key Laboratory of Medical Immunology Peking University Beijing 100191 China

**Keywords:** ceramide, HIF2α, obesity, pyrimidine, UMP

## Abstract

Metabolic abnormalities contribute to the pathogenesis of obesity and its complications. Yet, the understanding of the interactions between critical metabolic pathways that underlie obesity remains to be improved, in part owing to the lack of comprehensive metabolomics studies that reconcile data from both hydrophilic and lipophilic metabolome analyses that can lead to the identification and characterization of key signaling networks. Here, the study conducts a comprehensive metabolomics analysis, surveying lipids and hydrophilic metabolites of the plasma and omental adipose tissue of obese individuals and the plasma and epididymal adipose tissue of mice. Through these approaches, it is found that a significant accumulation of ceramide due to inhibited sphingolipid catabolism, while a significant reduction in the levels of uridine monophosphate (UMP), is critical to pyrimidine biosynthesis. Further, it is found that UMP administration restores sphingolipid homeostasis and can reduce obesity in mice by reversing obesity‐induced inhibition of adipocyte hypoxia inducible factor 2a (Hif2α) and its target gene alkaline ceramidase 2 (*Acer2*), so as to promote ceramide catabolism and alleviate its accumulation within cells. Using adipose tissue *Hif2α*‐specific knockout mice, the study further demonstrates that the presence of UMP can alleviate obesity through a HIF2α‐ACER2‐ceramide pathway, which can be a new signaling axis for obesity improvement.

## Graphical abstract

They conduct a comprehensive metabolomics analysis, surveying hydrophilic metabolites of the plasma and omental adipose tissue of obese individuals. They find that pyrimidine metabolism, especially uridine monophosphate (UMP), is significantly decreased in obesity. Furthermore, they demonstrate that the presence of UMP can alleviate obesity through a HIF2α‐ACER2‐ceramide pathway, which can be a new signaling axis for obesity improvement.

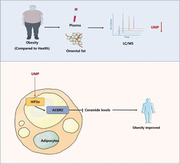

## 1 Introduction

Obesity is a global public health problem that is highly correlated with multiple metabolic diseases, such as diabetes, cardiovascular disease, nonalcoholic fatty liver, and several types of cancers.^[^ ^1^, ^2^ ^]^ Dysregulation of metabolic homeostasis that can arise from disorders in glucose uptake, fatty acid oxidation, and other energy‐producing or metabolic processes, can lead to cellular dysfunction and abnormal adipose tissue remodeling, and such characteristic changes are recognized as significant risk factors for obesity and its complications.^[^ ^3^ ^]^ Indeed, close monitoring of the dynamic changes of key metabolites and their signaling pathways can aid in the surveillance to maintain physiological homeostasis, to understand the underlying signaling mechanisms, and to detect and even prevent obesity and its complications.^[^ ^4^ ^]^

The presence and relative abundance of endogenous metabolites can be indicative of the physiological and pathological state of the body. For example, previous studies in animals and inhuman subjects have reported that changes to several metabolites were related to obesity, including branched‐chain amino acids (BCAAs), bile acids, aromatic amino acids, and fatty acid derivatives.^[^ ^5^, ^6^, ^7^, ^8^, ^9^ ^]^ However, current metabolomics studies have focused on establishing a correlation between changes to metabolites and diet or obesity, with an emphasis on the variation of the levels of lipids.

Obesity is known to be associated with a disorder of lipid metabolism, and sphingolipids represent one key type of lipid that plays an important role in the onset and progression of many metabolic diseases.^[^ ^10^, ^11^, ^12^ ^]^ Indeed, a disorder in sphingolipid metabolism that is observed during the development of obesity in mammals is known to lead to further complications.^[^ ^10^ ^]^ Ceramide is the central molecule of sphingolipid metabolism and the key node of sphingolipid de novo synthesis, rescue synthesis, sphingomyelin decomposition, and other pathways.^[^ ^13^ ^]^ Our previous work has demonstrated that a dysregulation of ceramide is central to the development of obesity and its related metabolic diseases.^[^ ^11^, ^14^, ^15^ ^]^ Ceramide dysregulation occurs ahead of systemic lipid metabolism abnormality, and acts as a key mediator in mediating organ cross‐talk to induce or aggravate obesity, atherosclerosis, diabetes, nonalcoholic fatty liver disease, and other metabolic diseases.^[^ ^14^, ^16^, ^17^ ^]^ Also, ceramide levels have been shown to be regulated by the functions of cellular transcription factors, including hypoxia‐inducible factor 2α (HIF2α), however, the underlying mechanisms in obesity is unclear. Furthermore, our understanding of how other factors, such as hydrophilic metabolites influence physiological homeostasis in the context of obesity is poorly characterized.

Here, we performed a comprehensive functional metabolomics analysis, covering both lipid and hydrophilic metabolomes (which we collectively define as “hydrophilomics”), to investigate whether changes to the metabolic profiles of humans and mice are causative for obesity. We show evidence of changes to sphingolipid and pyrimidine metabolism in obese subjects, including a significant decrease in the levels of uridine monophosphate (UMP) that is associated with obesity. Furthermore, we demonstrate that supplementation of UMP could ameliorate obesity through a mechanism involving restoration of pyrimidine and sphingolipid homeostasis and ceramide levels. Also, we conduct experiments with genetically modified *Hif2α* ^△adipo^ mice to show that *Hif2α* and its downstream target gene, alkaline ceramidase 2 (*Acer2*), are activated by UMP and this triggers ceramide decomposition, leading to reductions in ceramide levels and obesity traits. Taken together, we find that UMP levels can influence obesity through the restoration of metabolic homeostasis via a HIF2α‐ACER2‐ceramide signaling axis that is relevant for metabolic homeostasis and obesity.

## 2 Results

### 2.1 Sphingolipids are the Most Distinctly Varied Lipids in Obesity

Metabolic disorders are closely related to the onset of obesity.^[^ ^3^ ^]^ To investigate the characteristics of physiological metabolic homeostasis under obesity, we collected plasma and omental fat samples from eight obese volunteers (BMI > 30) and eight healthy volunteers (BMI < 25) from the General Surgery Department of the Third Hospital of Peking University (details in Table S1, Supporting Information), and analyzed both the lipophilic and hydrophilic metabolite properties in each sample.

**Figure 1:**
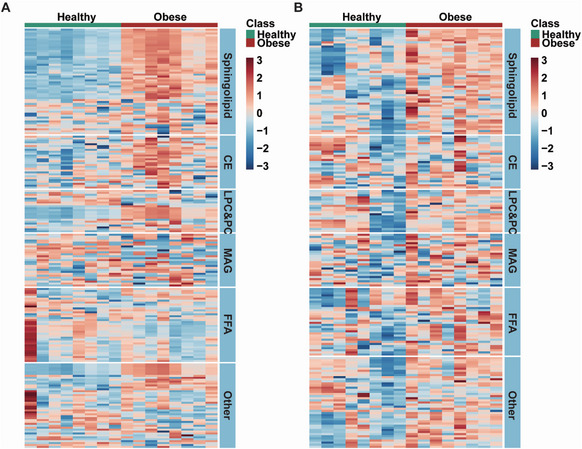
Sphingolipids are significantly differentiated in plasma and fat samples of obese individuals. Lipidomics detection and analysis were performed from plasma and fat tissue samples of healthy and obese individuals. A) Clustering heatmap of lipids in plasma. B) Clustering heatmap of lipids in fat. N = 8.

Lipophilic and hydrophilic metabolites in the plasma and fat samples were separated by the classic Folch extraction method^[^ ^18^ ^]^ and detected with LC‐MS/MS. For the lipidomics analysis, we found that the lipid profile of the plasma (**Figure** **1**A) and fat samples (Figure 1B) from obese individuals were significantly different from healthy individuals. Notably, the levels for sphingolipids were the most significantly different to other lipid components, both in terms of their subtypes and levels. Next, we generated obese mice models using a high‐fat diet (HFD) (see Experimental Section for details). As shown, using our protocol, we observed alterations in body weight, liver weight, epididymal fat weight, and subcutaneous fat weight in obese mice compared with controls (Figure S1A–D, Supporting Information), concomitant with increases in serum total cholesterol levels and triglyceride levels (Figure S1E,F, Supporting Information), as well as liver steatosis and lipid accumulation in the adipose tissue (Figure S1G, Supporting Information). Consistent with the results in obese human subjects, sphingolipids were also the most distinctly varied lipids in the lipidomics analysis of the plasma (Figure S1H, Supporting Information) and epididymal white adipose tissue (eWAT) (Figure S1I, Supporting Information) of obese mice compared to controls.

Given the diversity of the analytes among sphingolipids, we next performed a deep analysis on the sphingolipid metabolome to refine our results. Through this approach, our data showed that ceramides were markedly increased in obese individuals (Figure S2A,B, Supporting Information) and mice (Figure S3A,B, Supporting Information). It is recognized that ceramide is the key node in sphingolipid pathway that is involved in regulating the occurrence and development of obesity.^[^ ^13^, ^19^, ^20^ ^]^ To clarify the link between ceramide level and obesity, we performed correlation analysis between ceramide levels and mice body weight or human BMI index and found that ceramide levels were positively correlated with both mice body weight and human BMI index (Figure S4A,B, Supporting Information). Next, we looked into the regulatory mechanism of the sphingolipid pathway by determining the mRNA expressions of key enzymes in the eWAT of obese mice, and found that glucosylceramidase (*Gba 1/2*), an enzyme involved in salvage synthesis, as well as sphingomyelin phosphodiesterase (*Smpd3*), known to mediate sphingomyelin hydrolysis, were all activated, while the levels of ceramide decomposing enzyme alkaline ceramidase 2 (*Acer2*) was inhibited (Figure S4C–F, Supporting Information). These findings suggested that changes to the expression of such enzymes could underlie ceramide accumulation in obesity.

### 2.2 Pyrimidine Synthesis is Disrupted in Obesity

**Figure 2:**
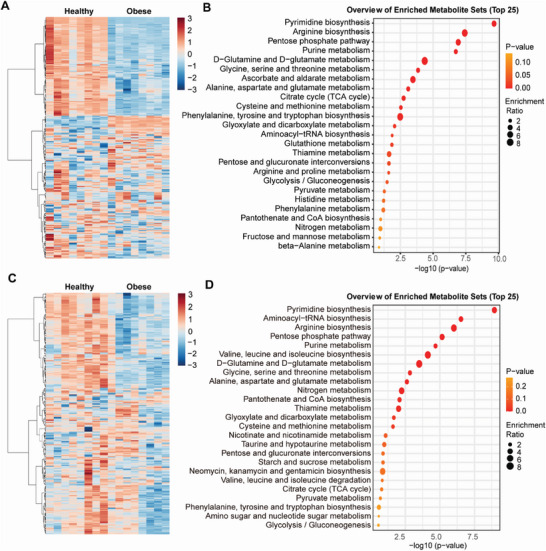
Hydrophilic metabolome is altered in obesity. Hydrophilomics detection and analysis were performed in plasma and fat tissues of healthy and obese individuals. A) Clustering heatmap of hydrophilomics in plasma. B) Enrichment analysis of hydrophilomics in plasma. C) Clustering heatmap of hydrophilomics in fat. D) Enrichment analysis of hydrophilomics in fat. N = 8.

The metabolic factors that drive lipid disorder and obesity are poorly characterized. To address this, besides lipidomics, we conducted comprehensive hydrophilomics. As shown, our plasma metabolomics data showed that the hydrophilic metabolites of obese people were markedly different from that of healthy controls, and extended beyond clear differences to levels of multiple lipid species (**Figure** **2**A). Next, we performed an enrichment analysis of metabolomic pathways and found that pyrimidine synthesis ranked as the most significantly modified pathways across plasma metabolomics profile (Figure 2B). Equally, our fat hydrophilomics analyses showed similar trends, with a clear distinction in the heatmap of metabolites between obese and healthy individuals (Figure 2C), and showing that pyrimidine biosynthesis ranked at the top in enriched metabolite sets (Figure 2D). Similar trends were also observed in HFD‐induced obese mice (Figure S5A–D, Supporting Information), altogether indicating a preeminent role for pyrimidine dysregulation in obesity.

**Figure 3:**
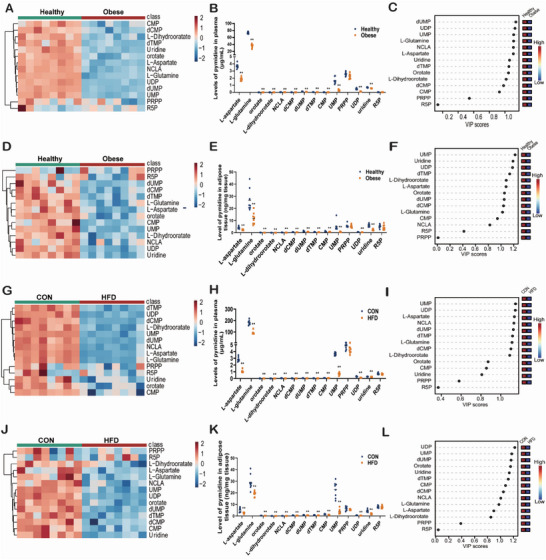
Pyrimidine metabolism is inhibited in plasma and fat tissues of obese humans and mice. Pyrimidine levels in plasma and fat tissues of obese individuals and mice were quantified (see Experimental Section for details). A) Pyrimidine clustering heatmap in human plasma. B) Pyrimidines quantification in human plasma. C) Pyrimidine VIP scores in human plasma. D) Pyrimidine clustering heatmap in human fat. E) Pyrimidine quantification in human fat. F) Pyrimidine VIP scores in human fat. G) Pyrimidine clustering heatmap in mice plasma. H) Quantification analysis of pyrimidines in mice plasma. I) Pyrimidine VIP scores in mice plasma. J) Pyrimidine clustering heatmap in mice eWAT. K) Quantification of pyrimidines in mice eWAT. L) Pyrimidine VIP scores in mice eWAT. All data are presented as the mean±SEM. Mann‐Whitney U test (B,E,H,K): ^**^P < 0.01, ^*^P < 0.05 versus CON, N = 8.

Guided by this insight, we next performed pyrimidine metabolome quantification studies to define key pyrimidine metabolites, using methods that we had previously reported.^[^ ^21^ ^]^ As shown, compared to healthy volunteers, analytes in pyrimidine synthesis pathways were markedly decreased in the plasma (**Figure** **3**A,B) of obese individuals. VIP scores analysis showed that UMP and uridine diphosphate (UDP) both ranked among the top three in the plasma samples (Figure 3C). Pyrimidines in the fat tissues of obese humans were also consumed (Figure 3D,E), with UMP ranking at the top in VIP scores (Figure 3F). Similar results were also observed in HFD‐induced obese mice (Figure 3G–L), and suggesting that UMP levels could be significant for the metabolomes of obese humans and mice.

### 2.3 UMP is a Critical Pyrimidine Metabolite in Obesity

**Figure 4:**
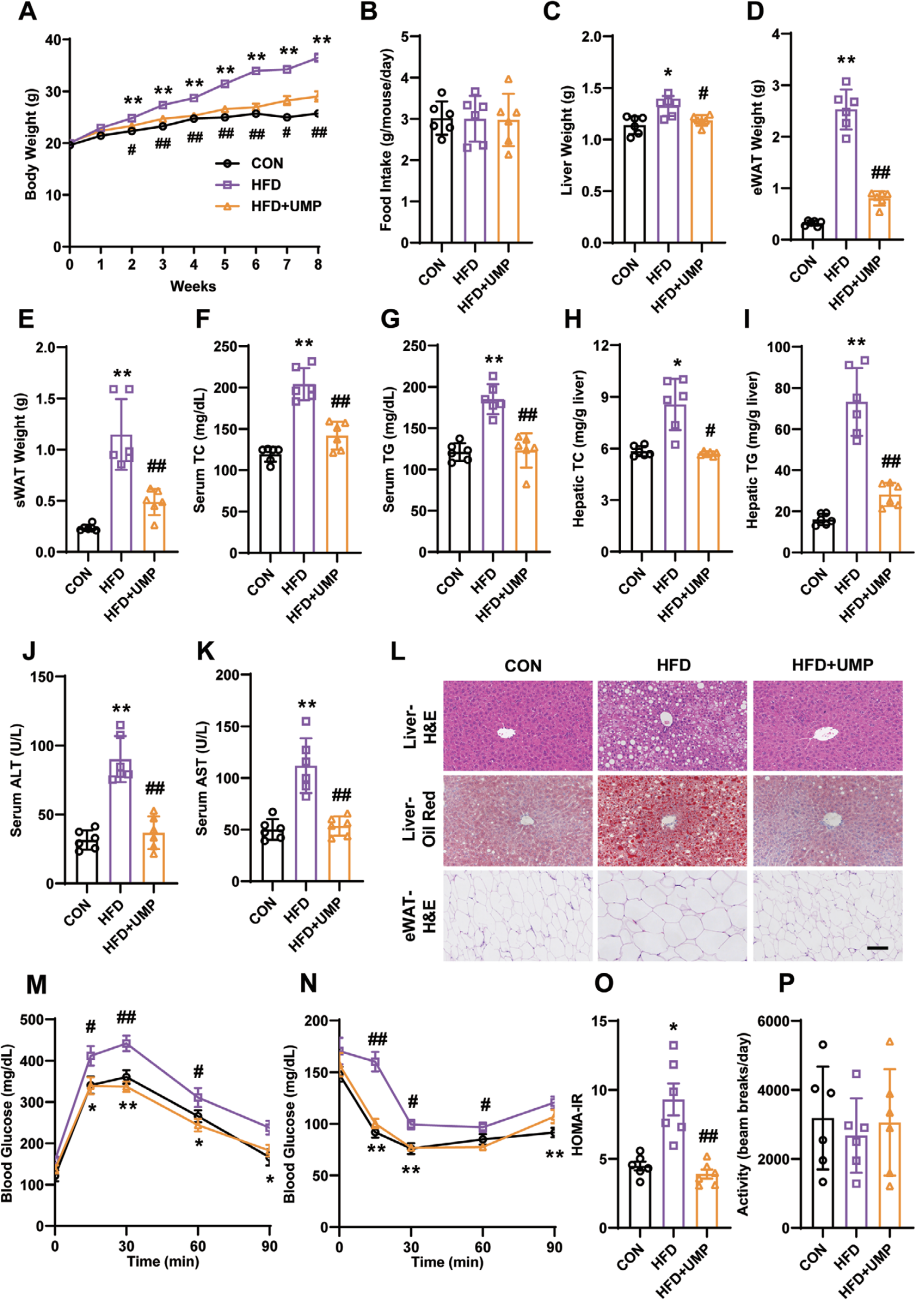
UMP supplementation alleviates obesity phenotype and improves energy metabolism in obese mice. Mice were divided into three groups, namely mice fed with normal chow diet (CON), high fat diet (HFD), and HFD combined with UMP in drinking water (HFD + UMP). A) Body weight. B) Food intake. C) Liver weight. D) Epididymal fat weight. E) Subcutaneous fat weight. F) Serum total cholesterol. G) Serum triglycerides. H) Hepatic total cholesterol. I) Hepatic triglycerides J) Serum alanine aminotransferase. K) Serum aspartate aminotransferase. L) H&E and oil red O staining of liver and eWAT, scale bar = 100 µm. M) Glucose tolerance curve. N) Insulin tolerance curve. O) Homeostatic Model Assessment for Insulin Resistance (HOMA‐IR) index. P) Exercise amount. All data are presented as the mean±SEM. one‐way ANOVA with Kruskal–Wallis test (A,B,E‐G,M,N), Dunnett's T3 post hoc test (C,D,H‐K,O) and Tukey's post hoc test (P): ^**^P < 0.01, ^*^P < 0.05 versus CON, *##P* < 0.01, *#P* < 0.05 versus HFD, N = 6.

UMP is the key node of the pyrimidine pathway and is the precursor for the synthesis of all other pyrimidines. Moreover, UMP links pyrimidine de novo synthesis with the salvage pathway, and initiates nucleic acid biosynthesis by transforming into UDP or uridine triphosphate, which is important for maintaining the physiological stability of pyrimidine.^[^ ^21^, ^22^, ^23^, ^24^ ^]^ Due to its significant role in the pyrimidine pathway and its prominence in obesity as a metabolite, as reflected in high VIP scores in our analyses (Figure 3), we next investigated the impact of modifying UMP levels as an approach to ameliorate obesity traits in mice. As shown in **Figure** **4**, supplementation of UMP (1 mg mL^−1^ UMP in drinking water) significantly alleviated HFD‐induced obesity, demonstrated as reductions in body weight, liver weight, and fat weight, and without affecting food intake in the mice (Figure 4A–E). Supplementation with UMP also decreased levels of total cholesterol and triglycerides in the serum and liver (Figure 4F–I), reduced serum ALT and AST (Figure 4J,K), improved liver steatosis and lipid accumulation in adipose tissue (Figure 4L), and normalized insulin resistance (Figure 4M–O), all without significant changes to the amount of exercise of obese mice (Figure 4P). These results suggest that obesity can be alleviated by modifying UMP levels through dietary supplementation, at least in mice.

### 2.4 UMP Alleviates Obesity by Restoring Pyrimidine and Sphingolipid Homeostasis

**Figure 5:**
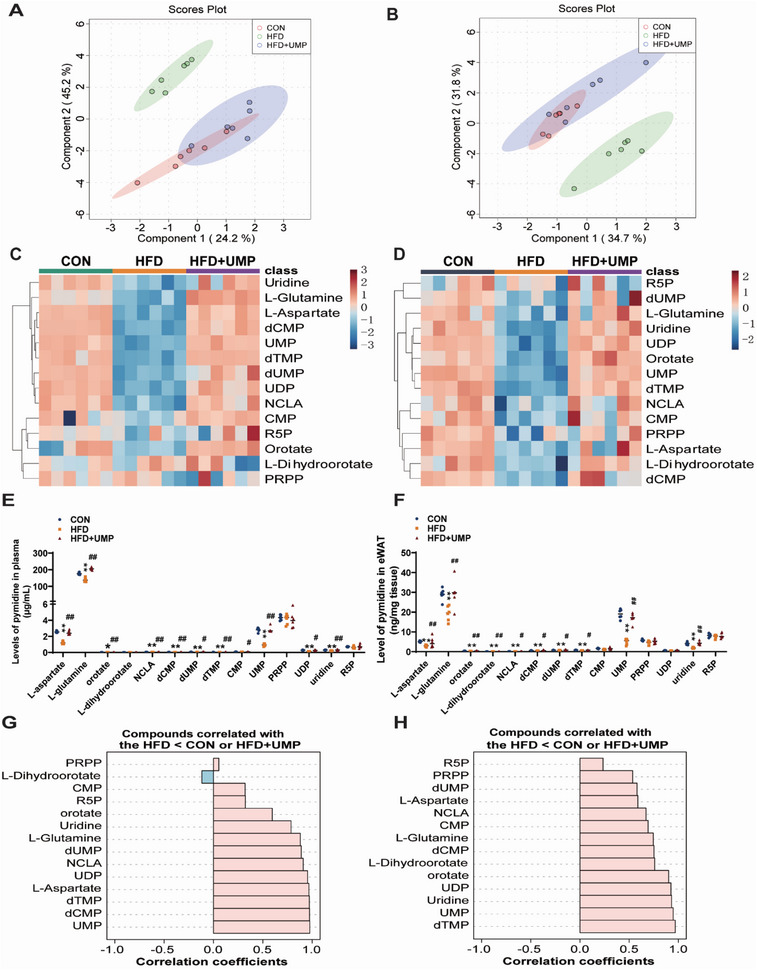
UMP supplementation restores plasma and eWAT pyrimidine homeostasis in obese mice. Pyrimidine omics were performed on plasma and eWAT of CON, HFD, and HFD + UMP mice. A) 2D PLSDA analysis of plasma. B) 2D PLSDA analysis of eWAT. C) Plasma pyrimidine clustering heatmap. D) Pyrimidine clustering heatmap of eWAT. E) Quantification of pyrimidines in mice plasma. F) Quantification of pyrimidines in mice eWAT. G) Plasma pyrimidine metabolites trend matching analysis, pink represents positive correlation, blue represents negative correlation. H) Pyrimidine metabolites trend matching analysis of eWAT, pink represents positive correlation, blue represents negative correlation. All data are presented as the mean±SEM. One‐way ANOVA with Kruskal–Wallis test (E,F): ^**^P < 0.01, ^*^P < 0.05 versus CON, *##P* < 0.01, *#P* < 0.05 versus HFD, N = 6.

**Figure 6:**
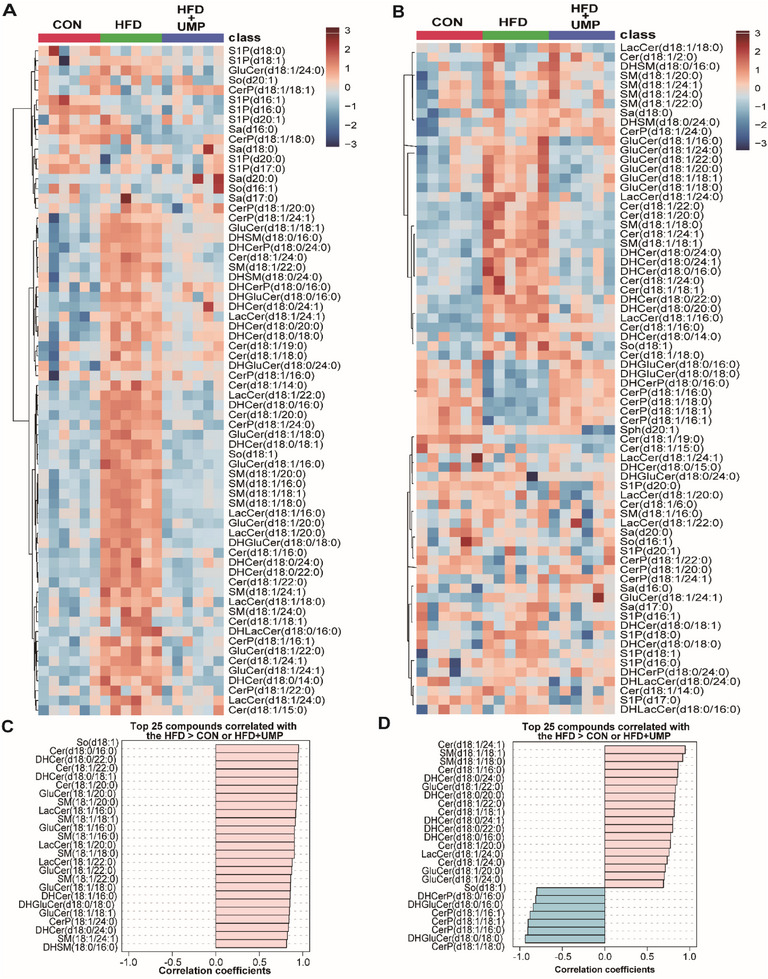
UMP Supplementation restores plasma and eWAT sphingolipid homeostasis in obese mice. Pyrimidine omics were performed on plasma and eWAT of CON, HFD, and HFD + UMP mice. A) Plasma sphingolipid clustering heatmap. B) Sphingolipid clustering heatmap of eWAT. C) Plasma sphingolipid metabolites trend matching analysis, pink represents positive correlation, blue represents negative correlation. D) Sphingolipid metabolites trend matching analysis of eWAT, pink represents positive correlation, blue represents negative correlation. N = 6.

To clarify the metabolic mechanisms underlying UMP‐induced alleviation of obesity traits, we analyzed the pyrimidine and sphingolipid metabolome of obese mice following UMP supplementation. Our Partial Least Squares Discriminant Analysis (PLS‐DA) of pyrimidine metabolomics showed that UMP supplementation was significantly restorative for obesity‐induced pyrimidine metabolic changes in both plasma (**Figure** **5**A) and eWAT (Figure 5B). As shown, our hierarchical clustering heatmap analyses demonstrated that UMP‐treated animals displayed metabolite characteristics reminiscent of controls, and were markedly different from samples from obese mice that were not supplemented with UMP in their drinking water (Figure 5C,D). Quantification analysis was conducted and showed that UMP supplementation reversed HFD‐induced reductions to the significant majority of pyrimidines detected in our analysis (Figure 5E,F). We also performed trend‐matching analysis and found that almost all pyrimidines had consistent positive trends in samples of obese mice with supplemented UMP (Figure 5G,H), further emphasizing the restorative role of UMP in this context. Furthermore, UMP supplementation corrected the defective sphingolipid profile of obese mice, and the levels of the significant majority of sphingolipids that were elevated in obese mice were significantly reduced in mice treated with UMP (**Figure** **6**A,B). In trend matching analysis, the top 25 sphingolipids in plasma and the top 18 compounds in eWAT were positively correlated with UMP supplementation, and the majority were ceramides and its derivatives (Figure 6C,D).

### 2.5 UMP Activates an Adipocyte HIF2α‐ACER2 Signaling Axis to Accelerate Ceramide Catabolism and Alleviate Obesity Traits

**Figure 7:**
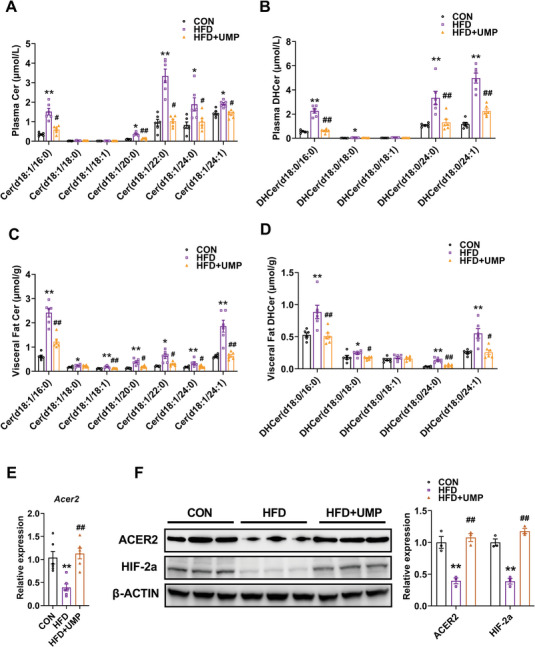
UMP restores ceramide levels by recovering HIF2α and ACER2 expressions. Ceramides in plasma and eWAT of CON, HFD, and HFD + UMP groups were quantified, and the expressions of HIF2α and its target genes were determined. A) Ceramide levels in plasma. B) Dihydroceramide levels in plasma. C) Ceramide levels in eWAT. D) Dihydroceramide levels in eWAT. E) *Acer2* mRNA expression in eWAT. F) *Hif2α* and its target gene *Epo/Dmt1* mRNA expressions in eWAT. G) ACER2 and HIF2α protein expressions in eWAT. All data are presented as the mean±SEM. One‐way ANOVA with Kruskal–Wallis test (A), Dunnett's T3 post hoc test (B‐D,F) and Tukey's post hoc test (E): ^**^ *P* < 0.01, ^*^ *P* < 0.05 versus CON, *##P* < 0.01, *#P* < 0.05 versus HFD, N = 6.

Given the relevance of ceramide to obesity and its significance in UMP‐mediated alteration of sphingolipids in our current findings, we quantified ceramide levels in obese mice using LC‐MS. The results showed that ceramide and dihydroceramide levels were significantly increased in plasma (**Figure** **7**A,B) and eWAT (Figure 7C,D) of obese mice, all of which were reversed by UMP supplementation. Also, we investigated the mRNA expression levels for key enzymes for ceramide synthesis and catabolism and found that only *Acer2* was significantly decreased in eWAT samples from obese mice, and that this reduction was alleviated by UMP supplementation (Figure 7E). In these samples, however, the expression of ceramide synthetic enzymes such as *Degs1*, *Gba1/2*, and *Smpd3* were not restored by UMP supplementation (Figure S6A–D, Supporting Information).

**Figure 8:**
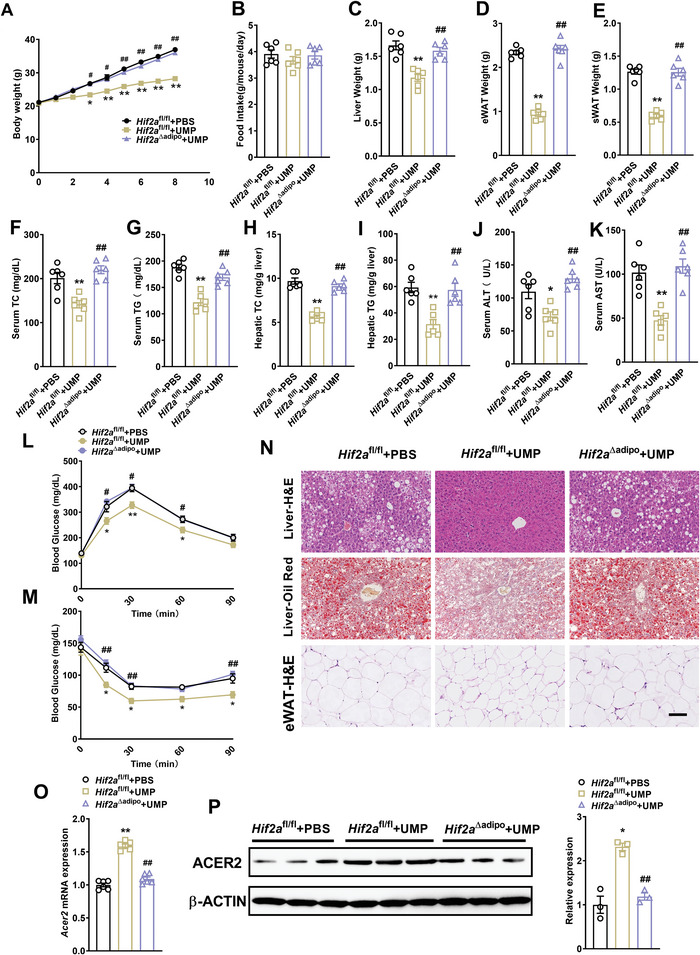
Adipose‐specific *Hif2α* knockout reverses UMP‐mediated obesity improvement. *Hif2α* ^fl/fl^ and *Hif2α* ^△adipo^ mice were treated with PBS or UMP‐drinking water, and obesity‐related biochemical indicators were determined. A) Body weight. B) Food intake. C) Liver weight. D) Epididymal fat weight. E) Subcutaneous fat weight. F) Serum total cholesterol. G) Serum triglycerides. H) Hepatic total cholesterol. I) Hepatic triglycerides. J) Serum alanine aminotransferase. K) Serum aspartate aminotransferase. L) Glucose tolerance curve. M) Insulin tolerance curve. N) H&E and oil red O staining of liver and eWAT sections, scale bar = 100 µm. O) *Acer2* mRNA expression. P) ACER2 protein level. All data are presented as the mean±SEM. One‐way ANOVA with Dunnett's T3 post hoc test(C‐G,I‐K,M,O,P), Tukey's post hoc test (B) and Kruskal–Wallis test (A,H,L): ^**^P < 0.01, ^*^ *P* < 0.05 versus *Hif2α* ^fl/fl^, ^##^ *P* < 0.01, *^#^P* < 0.05 versus *Hif2α* ^fl/fl^ + UMP, N = 6.

Our previous work confirmed *Acer2* as a target gene of hypoxia‐inducible factor (HIF).^[^ ^17^ ^]^ We detected the protein levels of HIF in eWAT, finding HIF1α activated (Figure S7A, Supporting Information) but HIF2α inhibited in obese mice (Figure 7F). Notably, we found that the inhibition of adipose HIF2α and its target gene levels in obesity could be alleviated by UMP supplementation (Figure 7F and Figure S7D, Supporting Information), but the activation of HIF1α and its target gene in obese mice were unaffected in this context (Figure S7B,C, Supporting Information), indicating that HIF2α was relevant to the capacity for UMP supplementation to alleviate obesity. To confirm this, we next developed an adipose‐specific *Hif2α*‐knockout mice model (Figure S8A,B, Supporting Information) and asked if genetic loss of HIF2α influenced UMP‐mediated alleviation of obesity. The data showed that UMP‐induced reduction of body weight, liver weight, fat weight, serum, and hepatic cholesterol and triglyceride levels, together with insulin resistance, liver steatosis relief, and lipid accumulation in adipose tissue were lost in *Hif2α* ^△adipo^ mice (**Figure** **8**A–N), and the upregulation of *ACER2* by UMP was also prohibited (Figure 8O,P). Taken together, our data suggests that UMP supplementation restores ceramide metabolism and improves obesity through a molecular pathway involving an HIF2α‐ACER2 signaling axis.

## 3 Discussion

Metabolic disorders accompany obesity and increase the risk of various metabolic diseases such as type II diabetes, cardiovascular disease, and non‐alcoholic fatty liver disease (NAFLD).^[^ ^2^, ^3^ ^]^ Metabolomics is a highly effective approach to simultaneously analyze both the types and relative quantities of the spectrum of endogenous metabolites within cells and tissues, and these data are indicative of metabolic homeostasis under specific physiological conditions.^[^ ^25^ ^]^ Previous obesity‐related metabolomics studies have focused on lipids,^[^ ^7^, ^8^ ^]^ and the abnormal metabolism of sphingolipids has been shown to be correlated with the onset and progression of obesity.^[^ ^10^ ^]^ Also, the concentrations of several kinds of medium and long‐chain sphingomyelin have also been demonstrated to be positively correlated with obesity and related metabolic indices.^[^ ^26^, ^27^ ^]^ As a key factor for sphingolipid metabolism, ceramide has been shown to be important to the progression of obesity.^[^ ^13^, ^14^, ^28^ ^]^ According to the previous report, exogenous administration of C16:0 ceramide can directly promote the expression of *Srebp1c* in the liver, inducing the exacerbation of non‐alcoholic fatty liver disease.^[^ ^29^ ^]^ At the same time, ceramide can directly induce hepatic endoplasmic reticulum stress, regulate hepatic gluconeogenesis, and aggravate hepatic lipid accumulation.^[^ ^30^ ^]^ Besides, ceramide could directly bind to FASN protein to promote excessive lipid synthesis. In our study, we found that sphingolipids were the most significantly altered class of lipids in obese humans and mice, a finding that is consistent with previous studies. Furthermore, we found that sphingolipid salvage synthesis and sphingomyelin hydrolysis were significantly activated, and ceramide decomposition was deficient, leading to an accumulation of ceramide. From our experiments, we found that UMP supplementation could alter ceramide levels and alleviate obesity‐induced metabolic traits, suggesting a potential application of UMP treatment in the context of mammalian obesity.

In addition to changes to the levels and types of lipids, several hydrophilic biomarkers were also described in the literature for obesity. For example, hydrophilic metabolites such as *L*‐kynurenine, glycerophosphocholine (GPC), glycerol 1‐phosphate, glycolic acid, tagatose, methyl palmitate, and uric acid were detected to be significantly altered between metabolic abnormal obesity and metabolic healthy obesity, based on a study for subjects from a central hospital in Taiwan.^[^ ^31^ ^]^ BCAAs levels were found to be significantly altered in the context of insulin resistance, which was reported to be caused by the synergistic inhibition of BCAA catabolic enzymes.^[^ ^32^ ^]^ Plasma glutamic acid was found to be increased in obese individuals, which was associated with intestinal the microbiota *Bacteroides multiforme*.^[^ ^33^ ^]^ Beta‐aminoisobutyric acid and 3‐hydroxyisobutryate were demonstrated to be correlated with obesity and exercise.^[^ ^34^, ^35^ ^]^ Despite such insight from previous studies, our understanding of the changes to hydrophilic substances and their potential impacts in obesity has been underdeveloped. Here, through our metabolomics approach that analyzed both the hydrophilic and lipophilic metabolomes, our hydrophilomics approach led to the identification of an underlying mechanism for the regulation of obesity traits in humans and mice, as follows. Our enrichment analysis identified pyrimidine synthesis as the most enriched metabolic pathway. This pathway is mainly responsible for the anabolism of pyrimidine nucleotides and plays a key role in tumor progression and neurotrophic effects.^[^ ^36^ ^]^ For example, dietary supplement of UMP or its downstream substance CDP can increase pyrimidine nucleotide in neurons and improve cognitive disorder, mood fluctuation, and behavior defects.^[^ ^37^, ^38^ ^]^ Dietary supplementation of uridine can significantly reduce body weight and improve liver lipid accumulation in HFD‐induced obese mice.^[^ ^39^ ^]^ Uracil‐supplemented diet increased locomotion in wild‐type mice via mitochondrial cristae formation in the liver, with protective effects against diet‐induced obesity.^[^ ^40^ ^]^ UMP is the key node substance in pyrimidine de novo and rescue synthesis, and also the source of other pyrimidine nucleotides.^[^ ^22^ ^]^ However, its role in obesity is still unknown. Our data in this study has demonstrated that exogenous supplementation of UMP, which is deficient in obesity, alleviated obesity traits through reductions in weight and lipid accumulation, reversing energy metabolism abnormalities, and improving insulin resistance and liver steatosis in obese mice.

Our metabolic mechanism study indicated that the beneficial effect of UMP on obesity was via restoring the physiological homeostasis of pyrimidine and sphingolipids, particularly, through rectifying the abnormal accumulation of ceramide. Our previous work found that as an important transcription factor, hypoxia‐inducible factor (HIF), mediates the occurrence and development of obesity and other metabolic diseases by regulating ceramide synthesis enzymes (SMPD3) or decompose enzymes (ACER2).^[^ ^14^, ^16^, ^41^ ^]^ Adipocyte HIF‐1α promotes ceramide synthesis by directly regulating *Smpd3* and aggravating atherosclerosis.^[^ ^42^ ^]^ Intestinal and adipocyte HIF2α transcriptionally activates *Acer2*, promotes ceramide decomposition, increases fat thermogenesis, and improves obesity.^[^ ^14^, ^41^ ^]^ In this study, we found that HIF1α was activated but HIF2α was inhibited in HFD‐induced obesity in mice. However, only the inhibition of HIF2α and its target gene *Acer2* was achieved by UMP supplementation in mice, and this was accompanied by the alleviation of ceramide accumulation and obesity features. Genetic loss of HIF2α in mice reversed UMP‐mediated *Acer2* activation and obesity alleviation, and we conclude from our findings that a HIF2α‐ACER2‐ceramide axis underlies the mechanism through which UMP supplementation can alleviate obesity features and restore metabolic traits. We speculate that targeting UMP and the HIF2α‐ACER2‐ceramide axis is relevant to the prevention and treatment of obesity.

## 4 Conclusion 

Pyrimidine synthesis is disrupted in obesity, within which UMP is distinctly depleted. UMP replenishment can alleviate obesity by recovering ceramide chaos in a HIF2α‐ACER2 signaling dependent mode.

## 5 Experimental Section

### Reagents and Materials

All standard analytes were purchased from Sigma Aldrich (St. Louis, MO) and were of analytical grade. Ultrapure water was prepared from a Milli‐Q integral water purification system (Millipore, Billerica, MA, USA).

### Studies in Humans and Animals

Human plasma samples were obtained from obese and healthy participants from Peking University Third Hospital who agreed to take part in the research. The experiments were carried out in accordance with The Code of Ethics of the World Medical Association (Declaration of Helsinki) for experiments and the research proposal was approved by the Ethics Committee of Peking University Third Hospital (No. IRB00006761‐M2021075). Detailed information about the volunteers is listed in Table S1 (Supporting Information).

SPF grade male C57BL/6J mice were purchased from Gempharmatech Co., Ltd and raised in a standard SPF environment. *Hif2a* ^fl/fl^ mice were generated as previously described.^[^ ^17^ ^]^ To achieve adipocyte‐specific disruption, *Hif2a* ^fl/fl^ mice were crossed with mice harboring Cre‐recombinase under the control of the adiponectin promoter (Adipoq‐Cre) to obtain *Hif2a* ^ΔAdipo^ mice.^[^ ^43^ ^]^ All studies were conducted according to the Guide for the Care and Use of Laboratory Animals (National Institutes of Health) and were approved by the Animal Care and Use Committee of Peking University (No. LA2023333). Male mice aged 6–8 weeks were randomly divided into a control (CON) group fed with maintenance diet, a high‐fat group (HFD) group fed with D12492 high‐fat diet, and a HFD + UMP group fed with high‐fat diet and 1 mg mL^−1^ UMP in water, for 8 weeks.

### GTT of Glucose Tolerance and ITT of Insulin Tolerance

In the mice glucose tolerance test, the mice were starved for 6 h and then intraperitoneally injected with glucose (2 g kg^−1^ body weight), and their blood glucose level was measured at 0, 15, 30, 60, and 90 min. In the insulin tolerance test, mice treated in the same batch were starved for 4 h and then intraperitoneally injected with insulin (2 IU/kg body weight), and their blood glucose level was measured at 0, 15, 30, 60, and 90 min.

### Detection of Energy Metabolism in Mice

The oxygen consumption and exercise volume were measured by using small animal metabolic cages. The mice were randomly placed in different metabolic cages, and the corresponding temperature and humidity (temperature 22 ± 2 °C, humidity 50 ± 10%), light cycle (12 h day/night), diet, and drinking water were completely consistent with those before the experiment. After adaptive feeding for 24 h, the oxygen and carbon dioxide levels in the metabolic cage were recorded continuously for 24 h, and the exercise volume and food intake were monitored at the same time.

### Determination of Total Cholesterol and Triglyceride

Plasma cholesterol and triglyceride levels were quantified using commercial kits (BioSino Bio‐Technology and Science, Beijing, China). Approximately 20 mg of liver tissue was weighed, homogenized in triglyceride cracking liquid, and centrifuged at 13 000 rpm for 10 min at 4 °C. The supernatant was collected to quantify hepatic cholesterol and triglyceride levels. Hepatic total cholesterol and triglyceride concentrations were quantified, normalized to the corresponding weight, and then expressed as milligrams of lipid per gram of tissue weight.

### Pathological Section Staining

Fresh tissues were excised, fixed in 4% paraformaldehyde, and paraffin‐embedded. Sections were stained with H&E. For detection of lipid, 7‐µm‐thick frozen cryosections of mouse liver were prepared from O.C.T‐embedded liver sections. Slides were fixed in 4% (v/v) paraformaldehyde for 1 hour, rinsed in ddH_2_O, and stained for 20 min in freshly prepared Oil Red O in 60% isopropanol.

### Fluorescent Real‐Time Quantitative PCR

Isolated RNA was treated with DNase using the Qiagen RNase‐Free DNase Kit and RNeasy Spin Columns (Qiagen, City, Country), and dissolved in RNase‐free water. The RNA quality was checked using the Agilent 2100 Bioanalyzer (Agilent Technologies, City, Country).

### Western Blot

The adipose tissues were lysed in RIPA buffer with protease and phosphatase inhibitors, the protein extracts were then resolved by SDS–PAGE electrophoresis and transferred onto polyvinylidene difluoride (PVDF) membranes. The membranes were blocked with 5% skimmed milk for 1 h at room temperature and then incubated with primary antibodies and HRP‐conjugated secondary antibody. The Anti‐ACER2 rabbit polyclonal antibody (Cat#PA5‐39016; RRID: AB_2555608) was from Invitrogen (California, USA). The anti‐HIF2α rabbit polyclonal antibody (Cat# NB100‐122; RRID: AB_10002593) was from Novus (Colorado, USA). The anti‐HIF1α rabbit polyclonal antibody (Cat#20960‐1‐AP, RRID: AB_10732601) was purchased from Proteintech (Rosemont, IL, USA). The anti‐β‐ACTIN mouse monoclonal antibody (Cat#A3854, RRID: AB_262011) was purchased from Sigma‐Aldrich (St. Louis, MO, USA). The blots were visualized with chemiluminescence, and the densitometry of the blots was detected using a LI‐COR Odyssey Fc imager (LI‐COR Biosciences, Lincoln, NE, USA).

### Metabolomics Analysis

For sample collection and preparation, 50 µL of plasma or 20 mg of fat tissue was added to 1 mL of extraction solvent (methanol/water 4:1 v/v), homogenized, oscillated for 10 min, and centrifuged at 14 000 g for 10 min at 4 °C. The supernatant was transferred, filtered through a 0.22 µm syringe filter (Shimadzu, Kyoto, Japan), and dried in a SpeedVac. The sediments were then dissolved in 100 µL of deionized water, and 5 µL was injected into the LC‐MS/MS system.

A QTRAP 5500 hybrid triple quadrupole/linear ion trap mass spectrometer (AB SCIEX, Concord, ON, Canada) connected to a UPLC system (Waters, Milford, MA) was used in hydrophilic or lipid metabolomics and pyrimidine pathway analysis. A Waters Xbridge amide column (100 mm × 4.6 mm i.d., 3.5 µm) at 40 °C was used in hydrophilic metabolome and pyrimidine pathway analyses, and a Waters UPLC BEH C18 column (100 mm × 2.1 mm i.d., 1.7 µm) at 40 °C was used in lipidomics analysis. The metabolite information and optimized mass parameters related to hydrophilomics, lipidomics, pyrimidine pathway, and ceramide pathway were consistent with previous reports.^[^ ^21^, ^44^, ^45^, ^46^ ^]^

### Statistical Analysis

Metabolomics analysis was carried out using the MetaboAnalyst 5.0 web service (http://www.metaboanalyst.ca/). Statistical analysis was performed using SPSS version 24.0, R version 3.2.4, and GraphPad Prism version 8.0.

Shapiro Wilk test was used for normality testing of all data. When it conforms to the normal distribution, comparisons between two groups were performed via two‐tailed unpaired Student's *t*‐tests, and comparisons between multiple groups were performed via ANOVA; when the data does not conform to the normal distribution, Mann Whitney U test was used to compare two groups of data, and Kruskal Wallis test was used to compare multiple groups of data. When ^**^P < 0.01, ^*^P < 0.05, ##P < 0.01 or #P < 0.05, it is considered that there is a significant difference.

## conflict of interest

The authors declare no conflict of interest.

## Author Contributions

L.H., W.P., and X.F. conceptualized and designed the study. L.H., W.P.,X.F., N.Q., and Y.S. performed the experiments and analyzed the data. Z.Y. and Z.Z. collected clinical samples. L.H., X.F., W.P., N.Q., and P.Y. wrote the manuscript with input from all of the authors. All of the authors edited the manuscript and approved the final manuscript. L.H., W.P., X.F., and N.Q. contributed equally to this work.

## Supporting information

Supporting Information

## Data Availability

The data that support the findings of this study are available from the corresponding author upon reasonable request.
