# Supplementary material for: The Hydrophilic Metabolite UMP Alleviates Obesity Traits through a HIF2α‐ACER2‐Ceramide Signaling Axis
Source: Adv Sci (Weinh). 2024 Mar 9;11(21):2309525. doi: 10.1002/advs.202309525 (PMC11151041; doi:10.1002/advs.202309525)
Supplement: Supplementary file 1 — Supporting Information [file ADVS-11-2309525-s001.pdf]

ADVANCED SCIENCE
Open Access

Supporting Information

for *Adv. Sci.*, DOI 10.1002/advs.202309525

The Hydrophilic Metabolite UMP Alleviates Obesity Traits through a HIF2α-ACER2-Ceramide Signaling Axis

*Huiying Liu, Pengcheng Wang, Feng Xu, Qixing Nie, Sen Yan, Zhipeng Zhang, Yi Zhang, Changtao Jiang, Xiaomei Qin and Yanli Pang**

Table S1 The baseline information about plasma and fat donators

Index	Health	Obese	P value
**Sex, Male/Female**	4/4	4/4	-
**Age, year**	48.25±0.13	45.38±0.21	0.486
**Height, cm**	167.00±0.05	168.38±0.06	0.775
**Weight, kg**	66.84±0.11	114.04±0.21	<0.001
**BMI, kg/m^2**	23.92±0.03	40±0.16	<0.001
**Systolic, BP, mmHg**	79.88±0.12	77.63±0.16	0.689
**Diastolic, BP, mmHg**	117.75±0.19	123.75±0.12	0.544

Data are presented as mean ± SEM. Two-tailed Student's t-test

Symbol: BP, blood pressure.

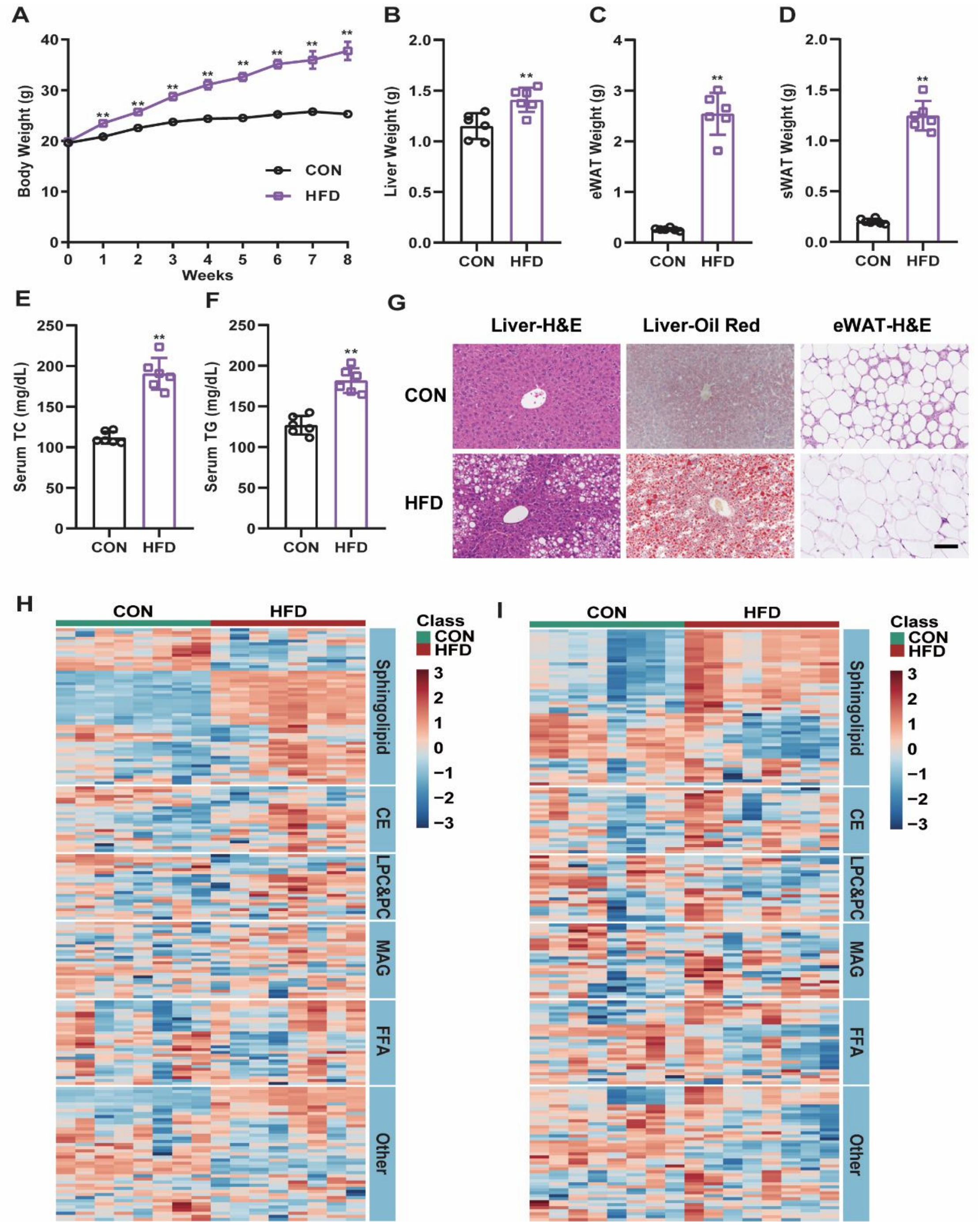

Fig S1. Sphingolipid is the most significantly differentiated lipid between obese mice and control. Mice were fed with chow diet (CON) or high-fat diet (HFD) to build obese mice model. (A) Body weight; (B) Liver weight; (C) Visceral fat weight; (D) Subcutaneous fat weight; (E) Total serum cholesterol; (F) Serum triglycerides; (G) H&E and oil red O staining of liver and adipose tissue sections, Scare bar=100 μm. (H) Clustering heatmap of lipids in plasma of mice. (I) Clustering heatmap of lipids in mice eWAT. All data are presented as the mean±SEM. Mann-Whitney U test (E) and Two-tailed Student's t-test (A-D, F): $^{**}P < 0.01$, $^{*}P < 0.05$ vs. CON, N = 6-8.

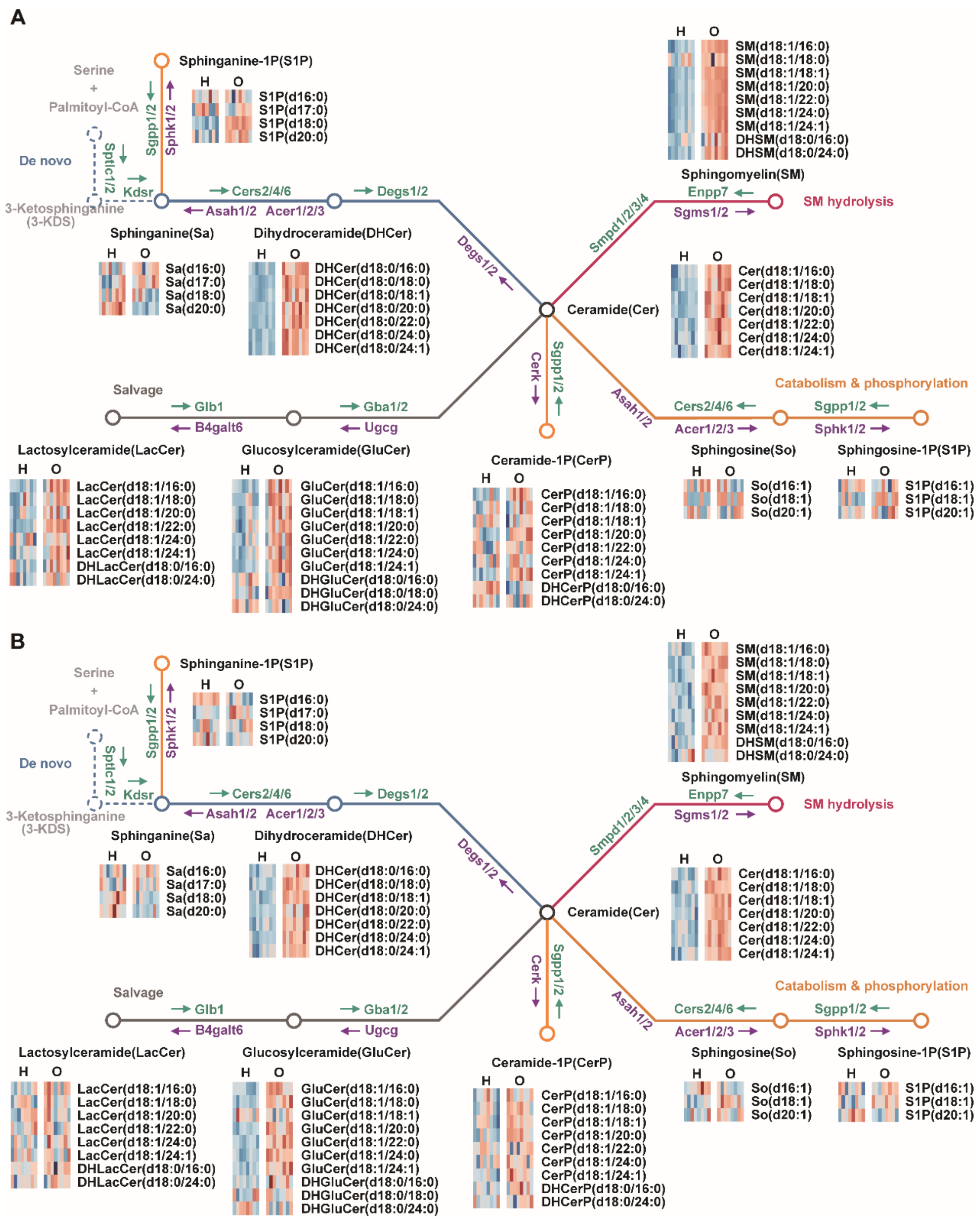

Fig S2. Ceramides are accumulated in plasma and fat of obese people. Sphingolipids were quantified in plasma and fat of obese people and a deep analysis was conducted. **(A)** Plasma sphingolipids spectrum; **(B)** Fat sphingolipids spectrum. N = 8.

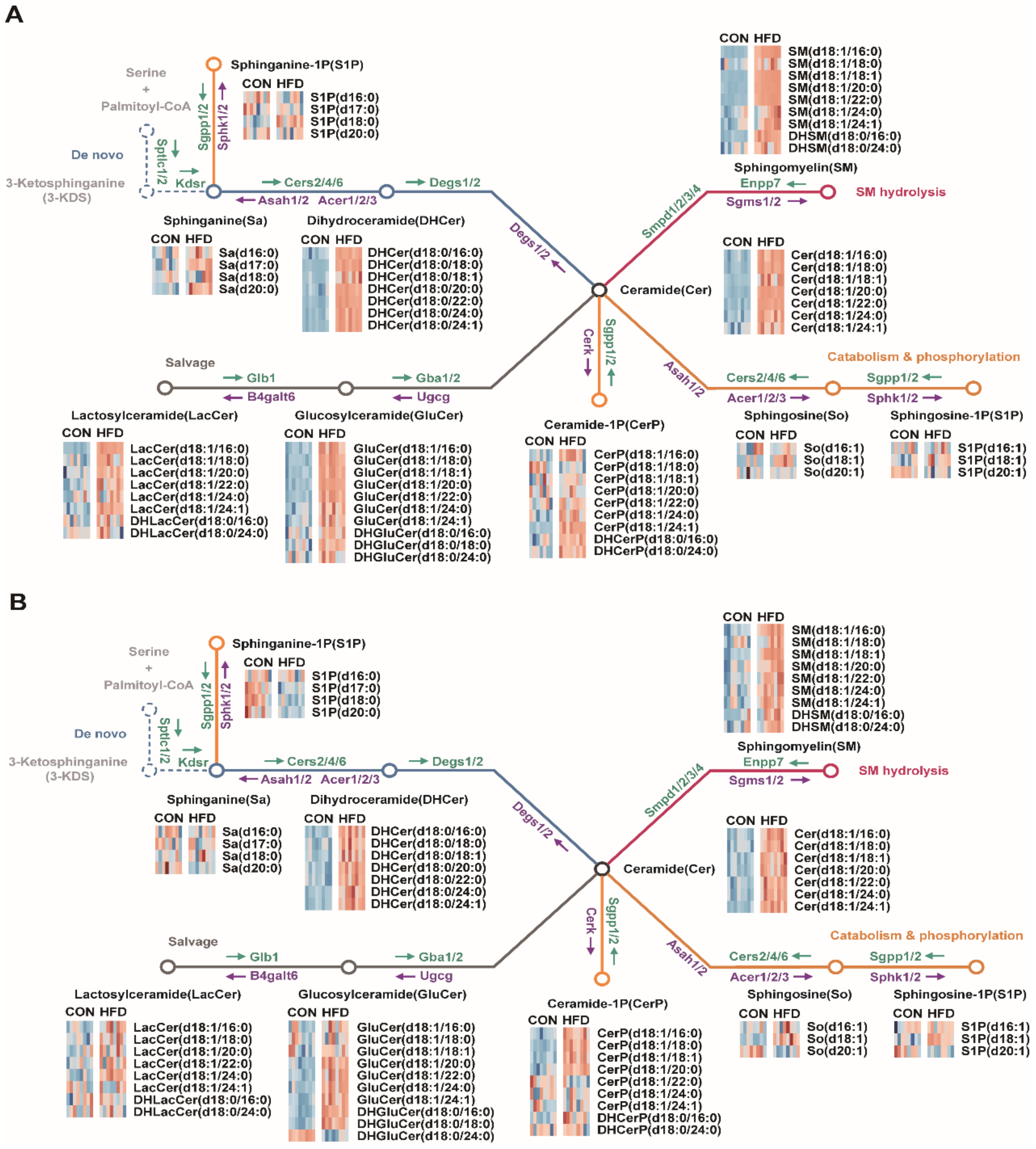

Fig S3. Ceramides are accumulated in plasma and fat of obese mice. Sphingolipids were quantified in plasma and adipose of obese mice and a deep analysis was conducted. **(A)** Plasma sphingolipids spectrum; **(B)** Adipose sphingolipids spectrum. N = 8.

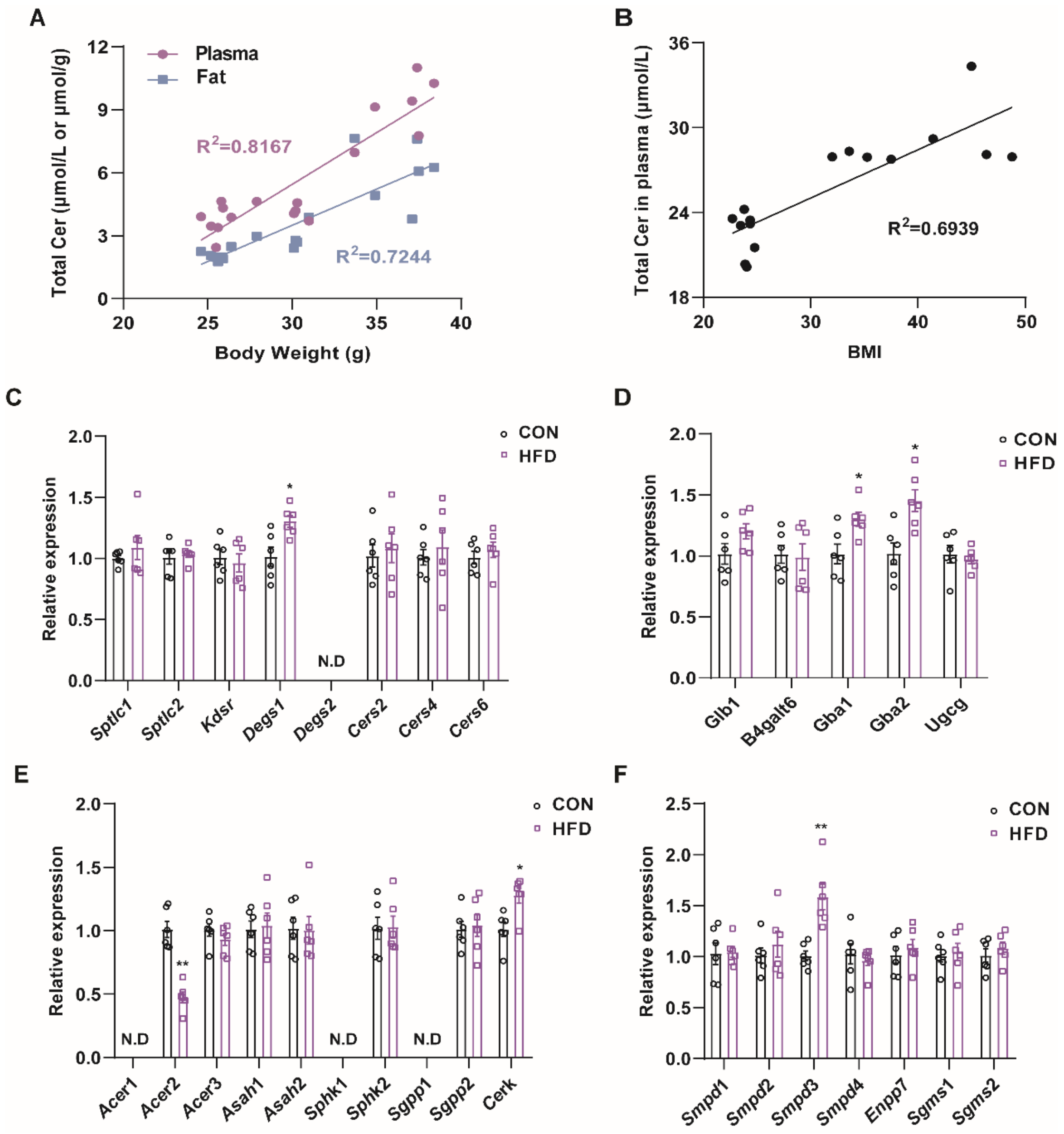

Fig S4. Ceramide levels are positively correlated with obesity and ceramide metabolism are regulated in obesity. (A) Correlation between plasma and fat ceramide levels with mice body weight. **(B)** Correlation between ceramide levels with human BMI index. **(C)** The mRNA expressions of enzymes in sphingolipids de novo synthetic pathway. **(D)** The mRNA expressions of enzymes in sphingolipids salvage synthesis pathway; **(E)** The mRNA expressions of enzymes in ceramide decomposition and phosphorylation pathways. **(F)** The mRNA expressions of enzymes in sphingomyelin decomposition pathway. All data are presented as the mean±SEM. Mann-Whitney U test (C, E) and Two-tailed Student's t-test (D, F): $^{**}P < 0.01$, $^{*}P < 0.05$ vs CON, N = 6.

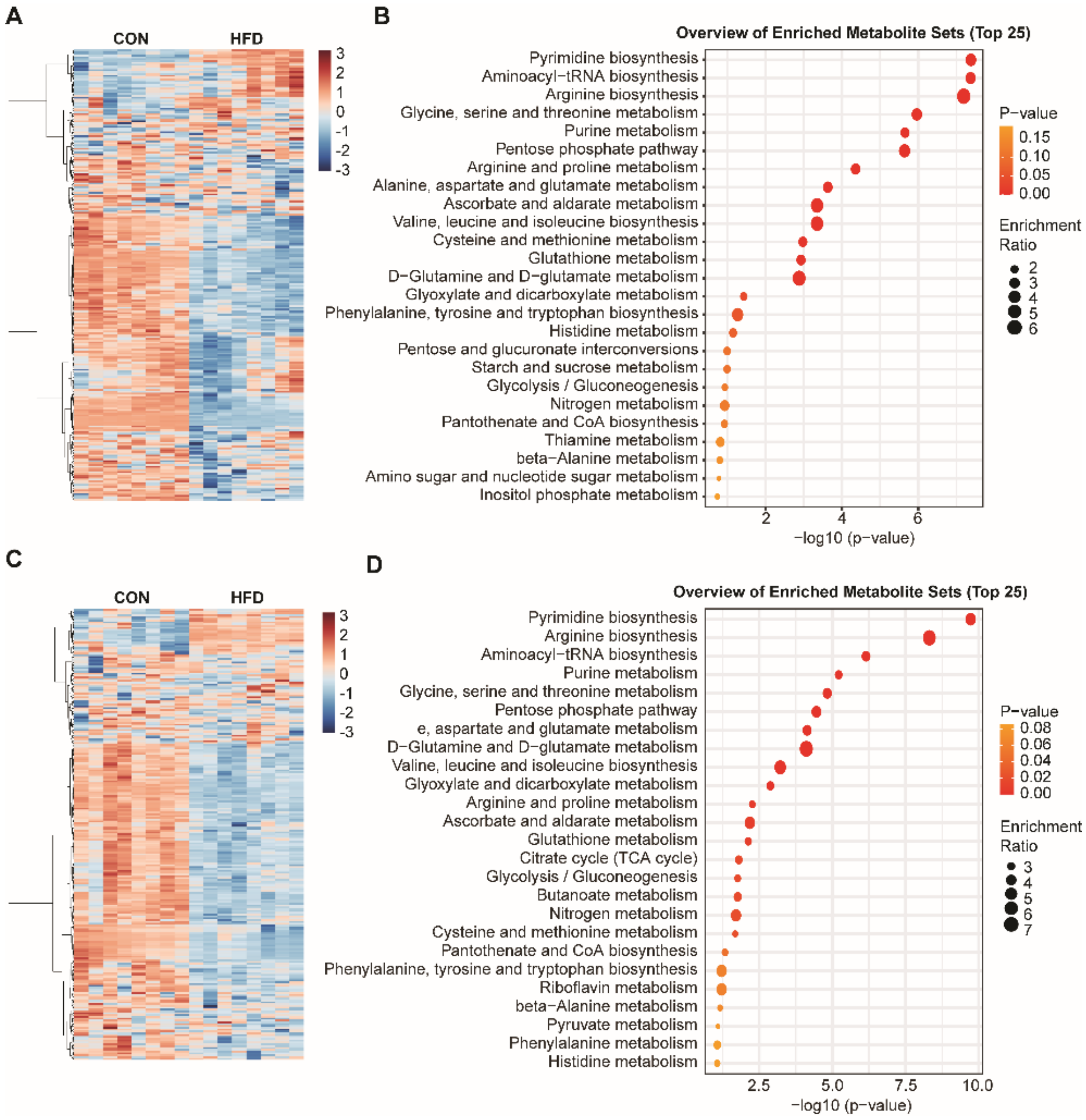

Fig S5. Hydrophilic metabolome varies between obese mice and control. Hydrophilomics detection and analysis were performed in plasma and eWAT in obese mice and control. **(A)** Clustering heatmap of hydrophilomics in mice plasma. **(B)** Enrichment analysis of hydrophilomics in mice plasma. **(C)** Clustering heatmap of hydrophilomics in mice eWAT. **(D)** Enrichment analysis of hydrophilomics in mice eWAT. N = 8.

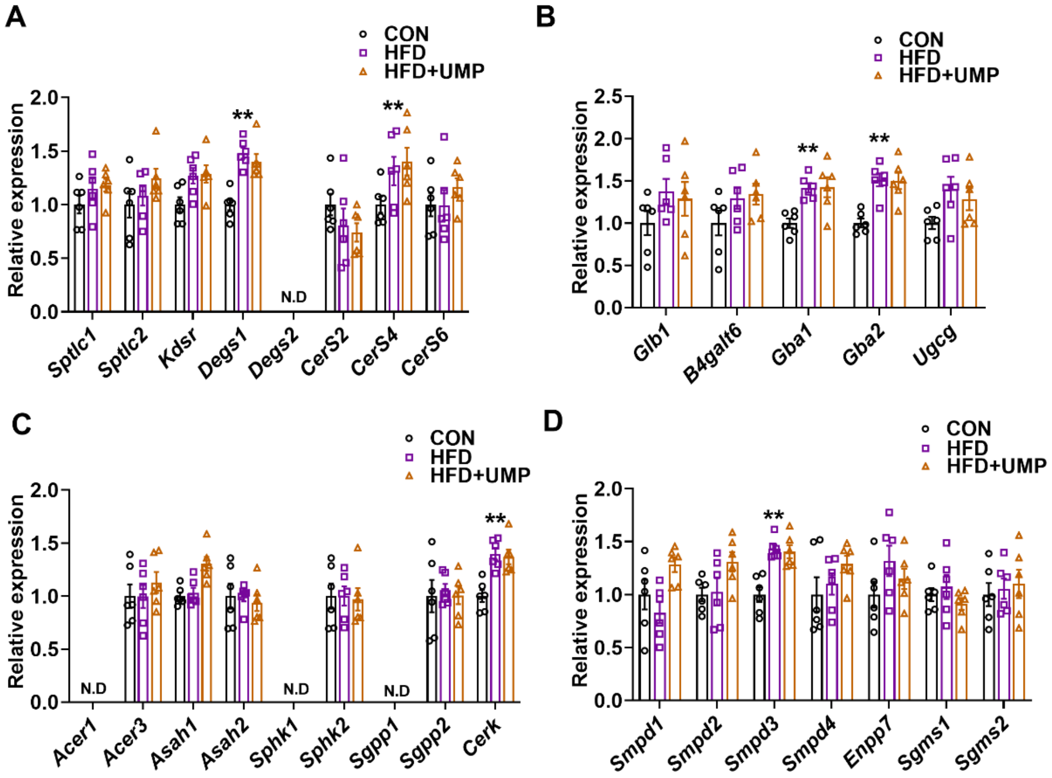

Fig S6. Change of most enzymes in ceramide metabolism were unaffected by UMP. The mRNA expressions of enzymes in **(A)** sphingolipids de novo synthesis pathway. **(B)** sphingolipids salvage synthesis pathway. **(C)** ceramide decomposition and phosphorylation pathways. **(D)** sphingomyelin decomposition pathway. All data are presented as the mean±SEM. one-way ANOVA with Kruskal-Wallis test (A-D): **$P < 0.01$ vs CON, N = 6.

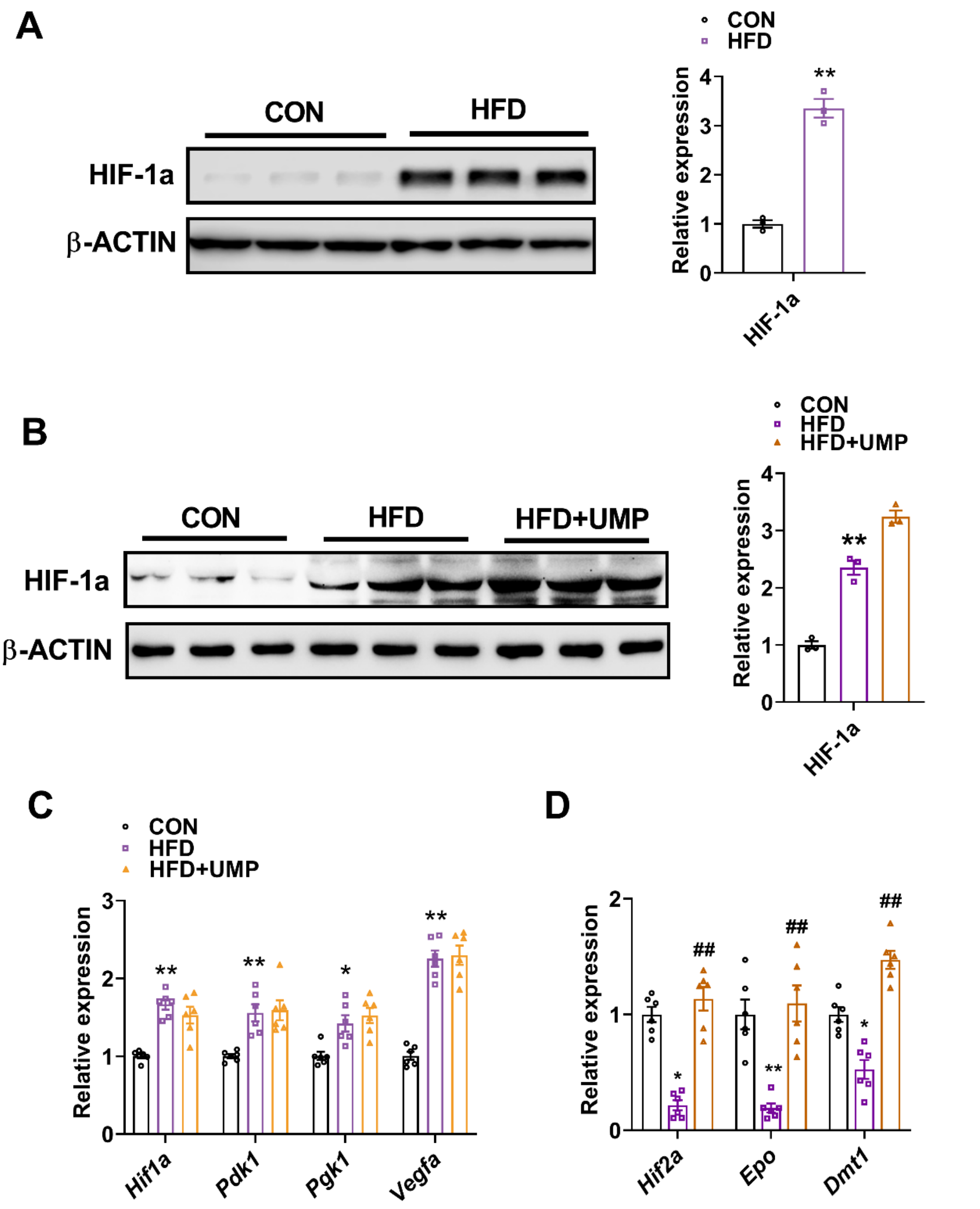

Fig S7. UMP treatment has no significant impact on obesity-induced HIF1α activation. (A) HIF1α protein levels in CON and HFD mice. **(B)** HIF1α protein levels in CON, HFD and HFD+UMP mice. **(C)** The mRNA expressions of *Hif1α* and its target genes. All data are presented as the mean±SEM. Two-tailed Student's t-test (A), one-way ANOVA with Dunnett's T3 post hoc test (B) and Kruskal-Wallis test (C, D): $**P < 0.01$, $*P < 0.05$ vs CON, N = 3 for protein levels and N = 6 for mRNA expression.

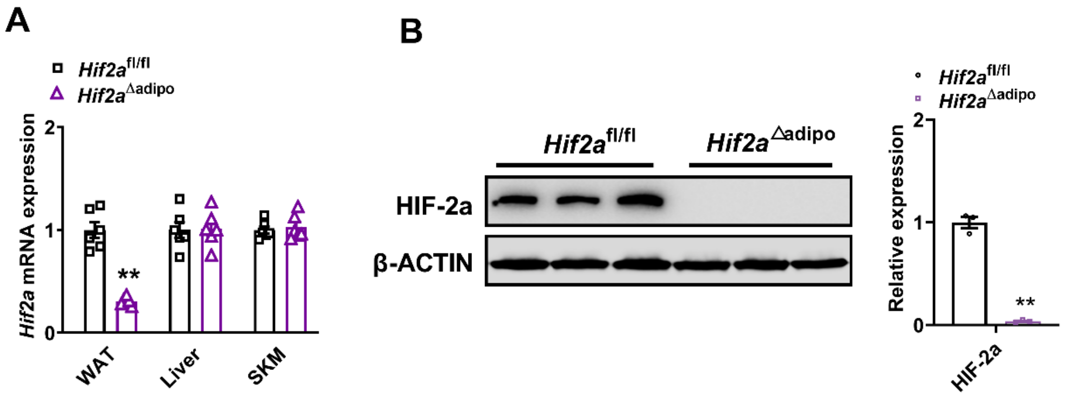

Fig S8. Knockout efficiency validation of *Hif2α* in *Hif2α*$^{fl/fl}$ and *Hif2α*$^{\Delta adipo}$ mice. (A) *Hif2α* mRNA level in eWAT, liver and skeletal muscle. **(B)** HIF2α protein level. All data are presented as the mean±SEM. Two-tailed Student's t-test (A, B): **$P < 0.01$ vs CON, N = 3 for protein levels and N = 6 for mRNA expression.
